# Supplementary material for: The Detection of Extensively Drug-Resistant Proteus mirabilis Strains Harboring Both VIM-4 and VIM-75 Metallo-β-Lactamases from Patients in Germany
Source: Microorganisms. 2025 Jan 25;13(2):266. doi: 10.3390/microorganisms13020266 (PMC11857796; doi:10.3390/microorganisms13020266)
Supplement: Supplementary file 1 [file microorganisms-13-00266-s001.zip › Supplementary Table S1.pdf]

Table S1. Predicted virulence factors in the genome of survcare401 as well as their presences in Survcare357 and Survcare372.

| # | Locus tag | Coordinates in Survcare401 (+/-) | Product | gene | Survcare372 | Survcare357 |
|---|---|---|---|---|---|---|
| 1 | *ORF1_236* | 263829..264767 (-) | ADP-L-glycero-D-mannoheptose-6-epimerase | *rfaD* | yes | yes |
| 2 | *ORF1_804* | 855013..855630 (+) | urease accessory protein (ureG) | *ureG* | yes | yes |
| 3 | *ORF1_1151* | 1276310..1276888 (+) | (gb\|NP_439337) (gmhA/lpcA) phosphoheptose isomerase | *gmhA/lpcA* | yes | yes |
| 4 | *ORF1_1181* | 1310618..1311133 (+) | (gb\|NP_230208) (luxS) S-ribosylhomocysteinase [AI-2 (VF0406)] | *luxS* | yes | yes |
| 5 | *ORF1_1504* | 1644629..1645678 (+) | Ureidoglycolate dehydrogenase | *allD* | yes | yes |
| 6 | *ORF1_1519* | 1666435..1668180 (+) | (gb\|NP_438233) (msbA) lipid transporter ATP-binding/permease | *msbA* | yes | yes |
| 7 | *ORF1_1549* | 1705594..1707072 (-) | type VI secretion system tubule-forming protein VipB | *vipB/mglB* | yes | yes |
| 8 | *ORF1_1551* | 1709491..1710009 (+) | type VI secretion system effector, Hcp1 family | T6SE | yes | yes |
| 9 | *ORF1_1552* | 1710093..1712285 (+) | type VI secretion system preotein VgrG | T6SE | yes | yes |
| 10 | *ORF1_1593* | 1758441..1759529 (-) | outer membrane protein A | *ompA* | yes | yes |
| 11 | *ORF1_1834* | 1998438..1999292 (+) | (gb\|NP_439706) (kdsA) 2-dehydro-3-deoxyphosphooctonate aldolase | *kdsA* | yes | yes |
| 12 | *ORF1_1862* | 2031526..2032044 (+) | type VI secretion system effector, Hcp1 family | T6SE | yes | yes |
| 13 | *ORF1_2130* | 2319691..2320269 (+) | superoxide dismutase | *sodB* | yes | yes |
| 14 | *ORF1_2220* | 2424837..2425742 (-) | (gb\|NP_438972) (galU) glucosephosphate uridylyltransferase | *galU* | yes | yes |
| 15 | *ORF1_2350* | 2576313..2577035 (-) | flagellar biosynthesis sigma factor | *fliA* | yes | yes |
| 16 | *ORF1_2363* | 2588834..2589832 (+) | flagellar motor switch protein G | *fliG* | yes | yes |
| 17 | *ORF1_2365* | 2590547..2591920 (+) | flagellum-specific ATP synthase FliI | *fliI* | not | yes |
| 18 | *ORF1_2369* | 2594475..2595506 (+) | (gb\|YP_001006748) (fliM) flagellar motor switch protein FliM | *fliM* | yes | yes |
| 19 | *ORF1_2370* | 2595499..2595909 (+) | (gb\|YP_001006749) (fliN) flagellar motor switch protein FliN | *fliN* | yes | yes |
| 20 | *ORF1_2372* | 2596359..2597129 (+) | (gb\|YP_001006751) (fliP) flagellar biosynthetic protein FliP | *fliP* | yes | yes |
| 21 | *ORF1_2373* | 2597144..2597413 (+) | (gb\|YP_001006752) (fliQ) flagellar biosynthetic protein FliQ | *fliQ* | yes | yes |
| 22 | *ORF1_2379* | 2602232..2603338 (-) | (gb\|YP_001006757) (flgI) flagellar P-ring protein precursor FlgI | *flgI* | yes | yes |
| 23 | *ORF1_2380* | 2603353..2604096 (-) | (gb\|YP_001006758) (flgH) flagellar L-ring protein precursor FlgH | *flgH* | yes | not |
| 24 | *ORF1_2381* | 2604159..2604941 (-) | (gb\|YP_106902) (flgG) flagellar basal body rod protein FlgG | *flgG* | yes | yes |
| 25 | *ORF1_2385* | 2607806..2608210 (-) | (gb\|YP_001006763) (flgC) flagellar basal-body rod protein FlgC | *flgC* | yes | yes |
| 26 | *ORF1_2386* | 2608216..2608629 (-) | (gb\|YP_001006764) (flgB) flagellar basal-body rod protein FlgB | *flgB* | yes | yes |
| 27 | *ORF1_2391* | 2612617..2614707 (-) | (gb\|YP_001006770) (flhA) flagellar biosynthesis protein FlhA | *flhA* | yes | yes |

| | | | | | | |
|---|---|---|---|---|---|---|
| 28 | *ORF1_2393* | 2616277..2616933 (-) | (gb\|YP_001006773) (cheZ) chemotaxis regulator CheZ | *cheZ* | yes | yes |
| 29 | *ORF1_2394* | 2616943..2617332 (-) | (gb\|YP_001006774) (cheY) chemotaxis regulatory protein CheY | *cheY* | yes | yes |
| 30 | *ORF1_2395* | 2617392..2618444 (-) | (gb\|YP_001006775) (cheB) chemotaxis-specific methylesterase CheB | *cheB* | yes | yes |
| 31 | *ORF1_2399* | 2623094..2623594 (-) | (gb\|YP_001006779) (cheW) purine-binding chemotaxis protein CheW | *cheW* | yes | yes |
| 32 | *ORF1_2400* | 2623616..2625835 (-) | (gb\|YP_001006780) (cheA) chemotaxis protein CheA | *cheA* | yes | yes |
| 33 | *ORF1_2402* | 2626923..2627792 (-) | (gb\|YP_001006782) (motA) flagellar motor protein MotA | *motA* | yes | yes |
| 34 | *ORF1_2403* | 2627951..2628532 (-) | (gb\|YP_001006783) (flhC) flagellar biosynthesis transcription activator FlhC | *flhC* | yes | yes |
| 35 | *ORF1_2404* | 2628535..2628885 (-) | (gb\|YP_001006784) (flhD) flagellar transcriptional activator FlhD | *flhD* | not | not |
| 36 | *ORF1_2472* | 2706620..2708074 (+) | (gb\|NP_273273) (katA) catalase | *katA* | yes | yes |
| 37 | *ORF1_2635* | 2895351..2895929 (-) | alginate biosynthesis protein AlgZ/FimS | *algU* | yes | yes |
| 38 | *ORF1_2857* | 3116799..3117719 (-) | (gb\|NP_439302) (lpxC) UDP-3-O-(R-3-hydroxymyristoyl) -N-acetylglucosamine deacetylase | *lpxC* | yes | yes |
| 39 | *ORF1_3055* | 3317204..3318469 (-) | Putative cytoplasmic protein | *ylbE* | yes | yes |
| 40 | *ORF1_3075* | 3336597..3337307 (-) | chaperone protein PapD | *papD* | yes | yes |
| 41 | *ORF1_3129* | 3400016..3400819 (-) | (gb\|NP_439219) (lpxA) UDP-N-acetylglucosamine acyltransferase | *lpxA* | yes | yes |
| 42 | *ORF1_3210* | 3503336..3504760 (-) | ADP-heptose synthase | *rfaE* | yes | yes |
| 43 | *ORF1_3335* | 3640480..3642126 (-) | Hsp60, 60K heat shock protein HtpB | *htpB* | yes | yes |
| 44 | *ORF1_3823* | 4174156..4174674 (+) | type VI secretion system effector, Hcp1 family | T6SE | yes | yes |
| 45 | *ORF1_3824* | 4174747..4176918 (+) | type VI secretion system preotein VgrG | T6SE | yes | yes |
| 46 | *ORF1_3827* | 4178188..4181232 (+) | type VI secretion system preotein IdsD | T6SE | yes | yes |
