# Supplementary figures and images for: The Detection of Extensively Drug-Resistant Proteus mirabilis Strains Harboring Both VIM-4 and VIM-75 Metallo-β-Lactamases from Patients in Germany
Source: Microorganisms. 2025 Jan 25;13(2):266. doi: 10.3390/microorganisms13020266 (PMC11857796; doi:10.3390/microorganisms13020266)

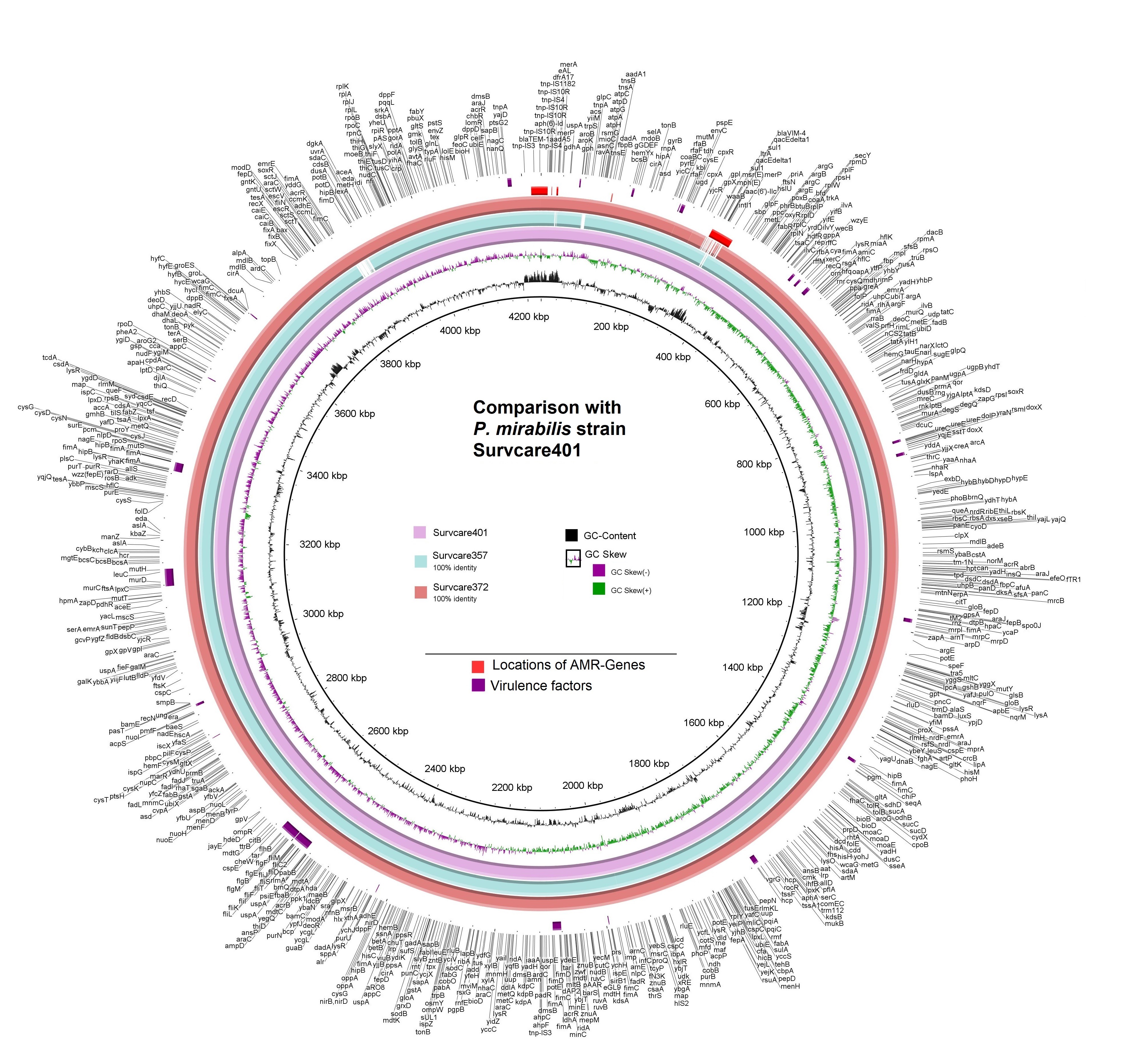

Supplement: Supplementary file 1 [file microorganisms-13-00266-s001.zip › Supplementary Figure_S1.jpg]
